# Practical Observations upon the Nature and Treatment of Prostatorrhœa

**Published:** 1860-07

**Authors:** S. D. Gross

**Affiliations:** Professor of Surgery in the Jefferson Medical College of Philadelphia


ART. III.—*Practical Observations upon the Nature and Treatment of Prostatorrhœa.* Read before the Medical Society of the State of Pennsylvania, in June, 1860. By S. D. GROSS, M.D., Professor of Surgery in the Jefferson Medical College of Philadelphia.

THE disease which I am about to describe has not, so far as is known to me, received any attention either from specialists or from the authors of general treatises on surgery. The term by which it is here designated appears, it is true, in M. Nélaton's "Elémens de Pathologie Chirurgicale," but altogether in an incidental manner, and evidently without any definite idea on the part of that distinguished writer as to the true nature of the affection in question.

Prostatorrhœa is, as the term implies, a discharge from the prostate gland, generally of a thin mucous character, dependent upon irritation, if not actual inflammation, of the component tissues of that organ. The reason why the disease has not hitherto received any specific name or place in surgical nomenclature, is simply because it has always been confounded with other lesions, as gleet, or chronic urethritis, seminal losses, and cystorrhœa, or chronic inflammation of the mucous membrane of the bladder; from which, in fact, it is often difficult to distinguish it. As for myself, I have long been familiar with the affection, and latterly have described it in my lectures at the College.

I have not met with prostatorrhœa in children or very young subjects, probably because all kinds of diseases of the prostate are so very rare at that period of life. That it may occur, however, even at a very tender age, is altogether likely, especially in children laboring under stone in the bladder, prolapse of the bowel, or worms in the rectum, causing the organ to suffer from reflected irritation. After the twentieth year the disease is sufficiently common, and instances are occasionally met with even in very old persons. As long as the prostate gland remains small and inactive, or is not brought fully under the influence of the sexual organs, with which it is so intimately associated, it is comparatively infrequent.

I am not able to say, from my experience, what classes of persons are most liable to suffer from this affection; but it has seemed to me that it is most frequent in those of a sanguineo-nervous temperament, with strong sexual propensities, leading to the frequent indulgence of the venereal appetite, if not to positive venereal excesses, either in the natural manner or by masturbation. An irritation would thus seem to be established in the prostate gland, attended with more or less discharge of its peculiar secretion, either in a normal or abnormal state. Single and married men are, apparently, equally prone to it. Once established, it is probable that certain occupations may serve to keep it up; and it is also likely that there are certain employments which may predispose to it, although it would require a much longer experience than what is possessed by any one individual to point them out in a definite or satisfactory manner. Intemperance in eating and drinking, frequent horseback exercise, sexual abuse, and disease of the bladder, anus, and rectum, may all be regarded as contributing to such a result.

The exciting *causes* of prostatorrhœa are not always very evident. In most of the cases that have fallen under my observation, the affection was traceable, either directly or indirectly, to venereal excesses, chronic inflammation of the neck of the bladder, stricture of the urethra, or disease of some kind or other of this canal. In some cases it has its origin in disorder of the lower bowel, as hemorrhoids, prolapse, fissure, fistule, ascarides, or the lodgment of some foreign body. It is easy to conceive how reflected irritation might induce this disease. The connection between the prostate gland and ano-rectal region is very close and intimate, and, hence, whatever affects the one will almost be sure, in time, to implicate the other, either in consequence of proximity of structure, or as an effect of the laws of sympathy. However this may be, no judicious surgeon ever omits to examine these parts most thoroughly in the event of any serious disease of any of them before he attempts a course of treatment. Temporary prostatorrhœa is occasionally excited by the exhibition of internal remedies, as drastic cathartics, cantharides, and spirits of turpentine; or, in short, whatever has a tendency to invite a preternatural afflux of blood to the prostate gland and neck of the bladder, or to the posterior portion of the urethra. Another cause of the disease, and, according to my experience, a very common one, especially in young men, is masturbation or self-pollution. Many of the most obstinate and perplexing cases of it that have come under my notice were the direct result of this detestable practice.

The *symptoms* of prostatorrhœa are sufficiently characteristic. The most prominent, as already stated, is a discharge of mucus, generally perfectly clear and transparent, more or less ropy, and of varying quantity, from a few drops to a drachm and upwards, in the twenty-four hours. It is seldom that it is puriform, and still more rare that it is purulent. When considerable, the flow keeps up almost a constant moisture at the orifice of the urethra, and may even make a decided impression upon the patient's linen, leaving it wet and stained, somewhat in the same manner as in gleet or gonorrhœa, though in a much less marked degree. The most copious evacuations of this kind generally occur while the patient is at the water-closet, engaged in straining, especially if the bowels are constipated, or the fæcal matter is uncommonly hard, or greatly distends the rectum, so as to exert an unusual amount of pressure upon the prostate gland.

The discharge, whether small or large, is often attended with a peculiar tickling sensation, referred by the patient to the prostate gland, from which it frequently extends along the whole length of the urethra, and even to the head of the penis. In some cases, indeed in many, the feeling is of a lascivious, voluptuous, or pleasurable nature, not unlike that which accompanies the earlier stages of sexual intercourse. Not a few patients experience what they call a "dropping sensation," as if the fluid fell from the prostate gland into the urethra. Other anomalous symptoms often present themselves, such as a feeling of weight and fatigue in the region of the prostate, the anus and rectum, or along the perineum, with, perhaps, more or less uneasiness in voiding urine, and a frequent desire to empty the bladder; some patients are troubled with morbid erections, and their sleep is interrupted with lascivious dreams.

It is astonishing how much the patient's mind often suffers in this affection. The discharge, even if ever so insignificant, occasions him the greatest possible disquietude; for at one time he imagines that it is a source of much bodily debility, or that it is productive of weakness and soreness in the dorso-lumbar region, especially if these symptoms happen to coexist; at another, that he is about to become impotent, under the delusive idea that the flow is one of a seminal character; an idea which not unfrequently haunts him day and night, and from which hardly anything can, perhaps, even temporarily divert his attention. His mind, in short, is poisoned, and the consequence is that he is incessantly engaged in trying to obtain relief, running from one practitioner to another, distrusting all, and affording none an opportunity of doing him any good. In the worst forms of the affection, his business habits are destroyed, he becomes morose and dyspeptic, and he literally spends his time in watching for the discharge which is the source and cause of his terrible suffering.

The affections with which prostatorrhœa may be confounded are the various forms of urethritis, especially gleet or chronic gonorrhœa, discharges of semen, and chronic inflammation of the bladder.

From urethritis, whether common or specific, it is generally easily distinguished by the history of the case, the nature of the discharge, and the attendant local phenomena. In most cases, the affection comes on gradually, not suddenly, as in gonorrhœa or simple inflammation, and without impure connection; the discharge is white or grayish, translucent, and ropy, not purulent, opake, and yellowish; and there is ordinarily no burning or scalding in micturition. Moreover, there is seldom any evidence of inflammation in the urethra or penis. In gleet or chronic urethritis the signs of distinction are sometimes more difficult; but even here a satisfactory conclusion may generally be reached by a careful consideration of the history of the case, and a proper examination of the discharge, which is nearly always more or less puriform, as well as more abundant than in prostatorrhœa. When the discharge of the urethra is kept up by the presence of a stricture, the diagnosis can be determined only by a thorough exploration with the bougie.

Very many patients confound this discharge with a flow of semen; an idea in which they are often encouraged by their attendants, in consequence of their ignorance of the nature of the affection. Much has been said and written respecting diurnal spermatic emissions; but, according to my experience, these evacuations are among the rarest occurrences met with in practice. We are often told that they take place at the water-closet, during efforts at straining, and this is, no doubt, occasionally the case; but more commonly it will be found that these discharges are of a strictly prostatic character, the fluid being forced out of its appropriate receptacles into the urethra, along which it is presently discharged. This delusion will be more likely to take hold of the mind if the escape of the fluid be accompanied by a sort of pleasurable sensation, somewhat similar to that which follows a feeble emission. Persons affected with prostatorrhœa will often tell us that they have quite a number of such evacuations—perhaps as many as six or eight—during the twenty-four hours, especially if they are troubled with disease of the ano-rectal region, leading to frequent visits to the water-closet, or if they are much in female society, engaged in exciting reading, or addicted to the pleasures of the table or to inordinate sexual intercourse, eventuating in general and local debility. Should the history of the case fail to afford the requisite light, it may be promptly supplied by a miscroscopic examination of the suspected fluid, semen always revealing distinct spermatozoa, whereas the prostatic and urethral secretions never afford any such indications. This will be the case whether the discharge be taken fresh from the orifice of the urethra or from the stiffened spots left upon the patient's linen.

The characteristic symptom of cystorrhœa, or chronic inflammation of the bladder, is an inordinate secretion of mucus, associated, in nearly all cases, with an altered condition of the urine, frequent and difficult micturition, pain in the region of the affected organ, as well as in the surrounding parts, and more or less constitutional disturbance. In prostatorrhœa there may also be more or less uneasiness low down in the pelvis, with trouble in voiding urine, especially where the prostate is much enlarged, so as to cause constant vesical irritation; but the two disorders are so widely different as to render it impossible to confound them.

The *pathology* of this affection consists in some disorder of the prostate gland, especially of its follicular apparatus, leading to an inordinate secretion of its peculiar fluid, and to a discharge of this fluid along the urethra, at longer or shorter intervals, and in greater or less quantity. That this disorder is, at times, of a real inflammatory nature, would seem extremely probable from the character of the concomitant phenomena, and also from the fact that this organ is frequently, if indeed not generally, found to be more or less enlarged and indurated. Nevertheless, there are cases, and these are by no means uncommon, in which it is, to all appearance, either entirely healthy, or so nearly so as to render it impracticable, by the most careful exploration, to discover any departure from the normal standard. The discharge under such circumstances seems to be the result solely of a heightened functional activity, probably connected with, if not directly dependent upon, disorder of the seminal vesicles, the urethra, neck of the bladder, or recto-anal structures; in other words, upon reflected irritation, or, as our professional forefathers would have denominated it, sympathetic disturbance.

The *prognosis* of prostatorrhœa is generally favorable; for it does not, in itself, present anything grave, being, as just stated, not a disease, but merely a symptom of a disease, usually slight, and therefore easily removable. Its obstinacy, however, is often very great, and hence the surgeon should always be guarded in the expression of his opinion respecting a rapid cure. When the mind deeply sympathizes with the local affection, as is so frequently the case, especially in young men of a nervous, irritable temperament, there is no disease which, according to my experience, is more difficult of management, or more likely to result in vexation and disappointment.

In the *treatment* of this affection, one of the first and most important objects is to inquire into the nature of the exciting cause, and, if possible, to remove it. To set about it in any other way would be the climax of absurdity; for here, as everywhere else, our therapeutic measures must be based upon a rational pathology, or a full appreciation of the nature and seat of the disease. The points which should more especially claim attention are, first, the condition of the prostate and its associate organs, and, secondly, the habits and state of health of the patient.

The first of these indications is best fulfilled by a thorough exploration of the genito-urinary apparatus and of the anus and rectum. For this purpose, a catheter is employed with a view of ascertaining the condition of the urethra, the prostate, and the bladder, aided by the finger in the bowel, previously emptied by an enema. In this manner, the surgeon becomes at once apprised of the existence or non-existence of stricture of the urethra, and of the presence or absence of morbid sensibility of its mucous membrane; the size and consistence of the prostate, and the state of the urinary reservoir, particularly as to whether there is inflammation, stone, hypertrophy, or other lesion. The finger in the rectum will be of great service, not only in detecting disease in the prostate and bladder, but also in this tube itself and in the anus. Indeed, without its aid no exploration of these organs could be at all satisfactory. If disease of the seminal vesicles exist, it will usually be evinced by tenderness on pressure through the wall of the bowel, provided the finger is sufficiently long or the prostate is not too voluminous.

The habits of the patient should be particularly inquired into. In many of this class of persons they are decidedly lascivious, or marked by excessive sexual indulgence, either naturally or in the form of masturbation, the prostate gland, seminal vesicles and adjoining structures being thus kept in a state of continual excitement, highly favorable to the production of prostatorrhœa. The nature of the patient's diet, his temperament, the state of his health, and his mode of life as it regards sleep and exercise, both of mind and body, also deserve special consideration.

Having ascertained the above facts, or, in other words, having made himself perfectly familiar with the local and general condition of the patient, the surgeon will be able, in most cases, to institute something like a rational mode of treatment. This should be directed, as a general rule, partly to the system at large, partly to the suffering structures.

In many of the cases the patient is weak, or deficient in muscular and digestive power, indicating a necessity for tonics, as iron and quinine, a nutritious diet, with a glass of generous wine, and gentle exercise in the open air, either on foot or in an easy carriage; riding on horseback being scrupulously avoided as likely to keep up undue excitement in the parts. One of the best preparations of iron is the tincture of the chloride, in union with tincture of nux vomica, in the proportion of twenty drops of the former to ten of the latter, four times a day. If the patient be plethoric, he may use with great advantage small doses of tartar emetic in the form of the antimonial and saline mixture, care being taken not to nauseate. In either case, it is of paramount importance to correct the secretions and to maintain a soluble condition of the bowels. Drastic purgatives are of course avoided, as they would only tend to perpetuate the mischief. Unless the patient is actually debilitated, he should rigorously abstain from condiments and high-seasoned dishes.

Among the more important topical remedies are, first, moderate sexual indulgence, as a means of allaying undue excitement of the prostate and its associate organs; secondly, cooling and anodyne injections, or weak solutions of nitrate of silver and laudanum, or, what I generally prefer, Goulard's extract with wine of opium, in the proportion of from one to two drachms of each to ten ounces of water, thrown up forcibly with a large syringe three times a day, and retained three or four minutes in the passage. In obstinate cases, cauterization of the prostatic portion of the urethra, or even of the entire length of this tube, may be necessary, the operation being repeated once a week. The cold hip-bath should be used twice in the twenty-four hours; the lower bowel should be kept cool and empty; and, if the disease do not gradually yield, leeches should be applied to the perineum and around the anus.

Such, in a few words, is a brief outline of the treatment which I have found most efficacious in this affection. Whatever plan may be employed, perseverance and an occasional change of prescription are indispensable to success. When there is deep mental involvement, hardly anything will effect a cure; or, more correctly speaking, it is almost impossible to induce the patient to believe that he is well, or that nothing serious is the matter with him. Under such circumstances our chief dependence must be upon travelling and an entire change of scene and occupation. If the patient be single, matrimony should be enjoined.

